# Controversies in the Anesthetic Management of Intraoperative Rupture of Intracranial Aneurysm

**DOI:** 10.1155/2014/595837

**Published:** 2014-03-03

**Authors:** Tumul Chowdhury, Andrea Petropolis, Marshall Wilkinson, Bernhard Schaller, Nora Sandu, Ronald B. Cappellani

**Affiliations:** ^1^Department of Anesthesia and Perioperative Medicine, 2nd Floor, Herry Medovy House, 671-William's Avenue, Health Sciences Center, University of Manitoba, Winnipeg, MB, Canada R3E 0Z2; ^2^Neurophysiology, Section Neurosurgery, Health Sciences Center, University of Manitoba, Winnipeg, MB, Canada R3E 0Z2; ^3^University of Southampton, Southampton S017 1 BJ, UK

## Abstract

Despite great advancements in the management of aneurysmal subarachnoid hemorrhage (SAH), outcomes following SAH rupture have remained relatively unchanged. In addition, little data exists to guide the anesthetic management of intraoperative aneurysm rupture (IAR), though intraoperative management may have a significant effect on overall neurological outcomes. This review highlights the various controversies related to different anesthetic management related to aneurysm rupture. The first controversy relates to management of preexisting factors that affect risk of IAR. The second controversy relates to diagnostic techniques, particularly neurophysiological monitoring. The third controversy pertains to hemodynamic goals. The neuroprotective effects of various factors, including hypothermia, various anesthetic/pharmacologic agents, and burst suppression, remain poorly understood and have yet to be further elucidated. Different management strategies for IAR during aneurysmal clipping versus coiling also need further attention.

## 1. Introduction

The incidence of unruptured aneurysms is progressively increasing worldwide [1, 2]. IAR remains a dreaded complication, with significant morbidity and mortality in affected patients [3]. Great strides have been made in the management of aneurysmal subarachnoid hemorrhage (SAH); however, outcomes have still not improved significantly [4]. This may be attributed to mechanisms such as early brain injury and delayed neurological ischemia, both of which can occur even with successful aneurysm clipping. The underlying pathophysiological mechanisms are not fully understood, and research is ongoing in this area [5, 6]. There is increased vulnerability to aneurysm rupture during the intraoperative period, and various challenges must be dealt with by perioperative physicians, including surgeons, neurointerventionalists, neuroanesthesiologists, and neurophysiologists. However, there is little data to guide the perioperative management of IAR, although intraoperative course may be the most important factor in determining overall neurological outcome. Furthermore, the literature mainly focuses on the management of aneurysm and SAH in toto. In this paper, we address the issues and controversies related to the management of IAR. Special reference is given to future directions in the management of such cases.

## 2. Methods

We performed a Pub Med, Scopus, Web Science, and Google search [1 January 1981 to 31 December 2012] using search terms including “Cerebral aneurysm,” “Intracranial aneurysm,” “Management,” “Anesthesia,” and “Intraoperative/Perioperative rupture.” All papers including prospective as well as retrospective studies and case series (minimum of 10 patients) in any language that specifically discussed the relevant management strategies in humans are included. Out of the 430 search results, 70 papers are included for this review.

*Exclusion*. Small series of patients (less than 10 patients), duplicated results, isolated case reports, and letters are not included. Articles related to management of pregnant patients, pediatric patients, and patients with complex giant aneurysms are not included in this review.

### 2.1. Causes of Intraoperative Rupture

Several studies have investigated multiple causative factors for rupture of intracranial aneurysms, though potentially detrimental intraoperative factors have not yet been fully elucidated [7–12]. However, it appears that IAR results from a complex interaction of etiological factors of aneurysm formation, as well as factors related to anesthesia, surgery, or other interventions (Table 1).

*Hypertension* is the most important risk factor for the formation of cerebral aneurysm, as well as aneurysm rupture [10–14]. Systemic hypertension increases the transmural pressure gradient (TMPG) and remodulates the vessel wall thickness. Patients with poorly controlled or labile blood pressure are generally considered to be at highest risk of rupture; however, IAR may also occur in patients with well-controlled hypertension. Intraoperative rupture can be precipitated by sudden fluctuations in TPMG, either due to high blood pressure or significant decreases in intracranial pressure (ICP). In previously normotensive patients, sudden hypertension may occur secondary to raised ICP but may also be associated with many other causes. The degree of hypertension at which aneurysm rupture is likely to occur is not presently known. Treatment of inadvertent hypertension should be prompt; however, caution is appropriate when aneurysm-associated hydrocephalus and increased ICP are present. However, TMPG cannot be the sole explanation for intraoperative aneurysm rupture. Several other factors are likely involved in aneurysm rupture, including thickness of aneurysm sac, type and location of aneurysm, type of surgical procedure, and intraoperative brain tension. There is some evidence to suggest that intraoperative hypertension is a significant cause for IAR; however, the presence of preexisting hypertension or high blood pressure due to various intracranial factors should also be taken into consideration when managing such patients [11–13].

Rarely, *induction of anesthesia* can also precipitate IAR (1-2% incidence). Rupture on induction portends very poor outcome, with a mortality rate up to 75% [15–17]. In a study of 404 patients undergoing aneurysm surgery, eight patients (2%) developed rupture at the time of induction and intubation [15]. Of these, six (75%) had anterior circulation aneurysms. Of the eight patients with aneurysm rupture, seven had either a complicated intubation or coughed during intubation, possibly indicating that airway manipulation and resultant sympathetic surge could have been a contributing factor [15]. Alternatively, these events could have represented rebleeding that simply coincided with induction. At present, data is inconclusive regarding the incidence of aneurysm rupture during conditions of stable induction of anesthesia and intubation. Perioperative hypertensive episodes can be observed during patient positioning, skull pin fixation, local anesthetic infiltration, skin incision, periosteal dissection, and dural opening, thus making the patient more vulnerable to intraoperative aneurysm rupture during these particular events. At the conclusion of surgery, extubation can again impose increased risk for aneurysm rupture or rebleed [18–20].

*Intracranial pressure* (ICP) may be increased in patients with poor grade aneurysm and is usually associated with worse outcome. Any sudden decrease in ICP will increase the TMPG and can hence produce IAR [21]. Decreased ICP may result from rapid mannitol administration or from hyperventilation prior to dural opening, although these variables are of primarily theoretical concern. Likewise, rapid CSF drainage via lumbar drains or ventriculostomy catheters could lead to IAR and may produce catastrophic consequences [20, 22]. Though there is little evidence to support ICP fluctuation as a major cause of IAR, it is advisable to decrease ICP slowly in the setting of known intracranial aneurysm.

Application of *certain maneuvers* like Valsalva and positive end expiratory pressure (PEEP) can also affect the transmural pressure and should hence be cautiously applied [20, 22].

*Comorbidities* such as coronary artery disease are linked with increased risk of IAR (odds ratio, 1.93 and 2.53, resp.) [9]. A possible explanation is that vessel wall strength may be altered in the presence of this disease. In addition, the presence of associated risk factors such as smoking and hypertension can also alter vessel wall fragility [9]. Preoperative assessment should include thorough evaluation of CAD and associated risk factors, as these comorbidities carry high risk for both cardiac events and cerebrovascular catastrophe.

In one study, *chronic obstructive pulmonary disease (COPD)* was found to be a risk factor associated with IAR, particularly in the coiling group [9]. Cigarette smoke (CS) is the most significant modifiable risk factor for cerebral aneurysm formation and also the major contributing factor related to COPD. Additionally, CS is a major risk factor for rupture with a hazard ratio reportedly as high as 3-4. The increased incidence of IAR in patients with COPD could be accounted for by smoking-related inflammatory changes, as well as other genetic and biochemical factors (alfa 1-antitrypsin deficiency and increased levels of matrix metalloproteinases) resulting in increased vessel wall fragility [9]. Ongoing inflammatory modulation, loss of vascular smooth muscle cells, decreased collagen synthesis, and excessive extracellular matrix breakdown likely all contribute to aneurysm rupture and SAH. It has also been proposed that intraluminal manipulation during coiling might lead to rupture in approximately 3% of the patients; however, the presence of increased airway resistance and its effect on TMPG in the closed cranium cannot be ignored [9].

### 2.2. Diagnosis of Intraoperative Rupture

Detection of IAR can be challenging (Table 2). However, during stable anesthetic conditions, a gradual unexplained increase in blood pressure along with a sudden decrease in heart rate is a common manifestation of IAR in both clipping and coiling procedures [15, 16, 23]. Sudden raised ICP and subsequent herniation can be manifested as a blown pupil, severe hemodynamic perturbations including arrhythmias, and ischemic signs on neurophysiologic monitoring (NPM).

Routine NPM commonly includes electroencephalogram (EEG), somatosensory evoked potential (SSEP), motor evoked potential (MEP), and brainstem auditory evoked potential (BAEP) monitoring [24].

*EEG.* Ischemia can be manifested by an initial transient increase in beta activity followed by the appearance of slow waves (theta and delta) with large amplitude. It is perhaps more common to see a decrease in the overall power spectrum of the EEG consistent with loss or weakening of alpha/beta frequencies and the predominance of, or loss of, low frequency components [24]. Ischemic events can progress to suppression of electrical activity with an occasional burst of activity (burst suppression) and finally to complete electrical silence with flat EEG, signaling the onset of irreversible damage. EEG is a sensitive marker for brain ischemia but cannot reliably determine the threshold between oligaemia and infarct as there is a variable range of cerebral blood flow below which tissue infarct occurs. It is also important to note that EEG is a cortical and as such is unable to assess the functional status of subcortical areas [24, 25].

*Evoked Potentials. *Cerebral ischemia slows neurotransmission and neuronal energy metabolism, resulting in decreased amplitude and increased latency of specific peaks. For SSEPs, a 50% reduction in amplitude and/or a 10% increase in latency [changes in the central conduction times, namely, the interpeak latencies between the N14 and N20 peaks] of SSEP signals from the baseline are generally accepted to be a significant change [26–29]. A 50% reduction on SEP amplitude has been shown to occur when cerebral blood flow decreases below 14 mL/100 g/min [30]. MEP have less well-defined warning criteria as compared to SSEPs; however, increased stimulus thresholds and/or decreased MEP amplitudes in relation to dramatic events (i.e., clip application) are indicative of pending neurologic insult. For BAEP, an increase in latency of more than 1 msec, particularly in wave V, is considered to be clinically significant. Unlike EEG monitoring the evoked potential tests can detect subcortical functional status by way of perforating branches such as the anterior choroidal and medial striate arteries [24].

EEG and evoked potential (EP) monitoring can be dramatically influenced by anesthetics and other physiological parameters including temperature and blood pressure [24, 25]. Thus there is a risk of erroneous interpretation and a failure to diagnose cerebral ischemia if these parameters are not maintained consistently. This underscores the importance of a coordinated strategy between anesthesiology and neurophysiology to provide the optimal conditions for neurologic monitoring. Some surgeons prefer the use of induced cerebral protection during periods of temporary clipping. Deepening the anesthetic level decreases the metabolic demand of neuronal tissue and increases the amount of time in which blood flow disruptions can be tolerated. To this end, burst suppression is frequently employed during temporary clipping. However, burst suppression essentially obviates the ability of these neurophysiological monitors to detect developing cerebral ischemia. EEG strength will once again recover after the period of burst suppression has ended; however, by this time ischemic changes are likely to be irreversible. This underscores the need to employ EEG/EP modalities in combination so that some measure of neurologic evaluation is maintained during periods of EEG suppression. The ability of SSEPs to detect deficits has been reported to be quite low [31]. However, if the neural territory that is assessed by SSEP is examined for ischemic lesions, the ability of this test to correctly predict ischemic events is high at 93% [32]. The inability of SSEP to detect infarcts outside the somatosensory pathway may explain why the use of SSEP has not been more widely adopted. This does not, however, represent a failure of the monitor; rather, it represents an inappropriate application. Similarly the recovery of SEPs after a loss of the potential, despite postoperative deficits, reflects the status of the somatosensory cortices and not other areas of the brain. There has been no data to our knowledge that assesses the ability of multimodality NPM to predict ischemic episodes. The incorporation of MEP allows the assessment of the corticospinal system and in combination with EEG and SSEP increases the ability to monitor important functional areas of the nervous system [24].

Other monitoring methods such as transcranial Doppler, ultrasound, and different devices to monitor cerebral oxygenation may be useful to detect intraoperative rupture, especially if rupture occurs before dural opening [33–35]. One report highlighted the significance of TCD for detection of IAR and the major finding was the loss of diastolic flow or even the reversal of diastolic flow [33]. Intraoperative ultrasonography is also used to diagnose and differentiate causes of brain bulge, including hematoma due to rupture of aneurysm and development of acute hydrocephalus [34]. This technique utilizes the special window (Paine's point) to provide axial images showing the anterior interhemispheric fissure, lentiform nucleus, insular cortex, Sylvian fissure, and ventricular system. On the other hand, other devices such as noninvasive and invasive cerebral oximetry have been employed mainly for detection of cerebral ischemia during clip placement; however, sudden reduction of values may be one of the earliest signs of IAR if other possibilities have been ruled out [35].

Intraoperative imaging modalities including CT, MRI, and different methods of angiography can be utilized for prompt diagnosis of IAR and other complications [36, 37].

In cases of surgical clipping, some authors have reported increased ooze during scalp incision and after craniotomy in the case of aneurysm rupture; unexpected marked brain bulge was seldom noticed [15, 16]. Even intraoperative brain bulge can sometimes be the only sign which predicts the subtle IAR [38]. Thus IAR should be considered as a possible differential diagnosis in the setting of unexpected tense brain if other causes have been ruled out [38].

Intraoperative aneurysm rupture during embolization is a potentially devastating event. The common contributing factors are mainly iatrogenic in nature and include guide wire or microcatheter-induced perforation, coil penetration, high-pressure contrast injection, and excessive packing of the coil material [23]. The common presentations during rupture usually comprise acute increase in systemic hypertension with bradycardia, dye extravasations, and prolongation of the contrast dye transit time [23, 39]. Significant increases in ICP can result in slowing or even flow arrest of the ICA and flow reversal to the external carotid artery. Intraoperative imaging modalities can usually detect this complication immediately; however, the overall mortality and morbidity remain high and need prompt management [23].

Postoperatively, delayed awakening, sudden deterioration of consciousness, changes in hemodynamic parameters, seizures, and focal neurological deficits may be signs of aneurysm rerupture [40].

## 3. Management

This section mainly focuses on the management of intraoperative rupture of intracranial aneurysm, depending upon the time of rupture intraoperatively. Rupture of an aneurysm with an open skull and dura carries a better prognosis than a rupture occurred in a closed skull; thus different management during these two different intraoperative phases is warranted [15, 16].

### 3.1. Rupture before Dural Opening

Intraoperative cerebral aneurysm rupture in a closed skull produces sudden increases in ICP, thus jeopardizing cerebral perfusion. The result is cerebral ischemia and ultimately irreversible neuronal injury [15, 16]. In this situation, management goals commonly include rapid ICP reduction as well as implementation of neuroprotective strategies [15, 16, 18, 19, 22]. However, rapid ICP reduction in the face of IAR remains controversial, as raised ICP may in fact reduce ongoing bleeding by means of a tamponade effect. Nonetheless, ICP reduction in this situation may be accomplished by means of intravenous anesthetics (propofol, thiopentone sodium), hypothermia, and hyperventilation [18, 19, 22]. Intravenous anesthetics may be the optimal choice as they can provide both a reduction in cerebral metabolism and a modest reduction in ICP via flow-metabolism coupling. Hypothermia may also afford some degree of neuroprotection in addition to reducing ICP; however, rapid implementation of hypothermia for IAR is largely not feasible. It is possible to initiate hypothermia during the predissection stages of surgery; however, the benefit of this intervention is unknown and further investigation is required before it can be recommended [20, 22]. Hyperventilation is also a potent therapy for prompt reduction in ICP; however, hyperventilation-induced vasoconstriction can worsen ongoing ischemia. Nevertheless, a short period of moderate to severe hyperventilation may be a reasonable rescue measure, and cerebral oxygenation monitoring may assist in the safe application of hyperventilation in this setting [18–20, 22].

Hemodynamic goals are poorly defined in the setting of IAR with a closed skull or dura, although a 20% reduction in blood pressure from baseline is commonly advocated in this situation [15, 16, 20, 22, 41]. Reductions in blood pressure, however, can lead to reduced cerebral perfusion and worsening cerebral ischemia. Prompt surgical intervention including rescue clipping is a key factor in determining the outcome of these cases [15].

Preprocedural rupture of cerebral aneurysm during embolization can have dire consequences, due to the presence of a closed cranium [42]. Prompt reversal of heparin anticoagulation, minimization of cerebral metabolism, and control of abrupt increases in ICP are the primary goals in this situation [23]. The use of intravenous anesthetic agents, immediate blood pressure control, and modest hyperventilation is the options which can be instituted promptly [20, 22]. Surgical intervention such as ventriculostomy or urgent craniotomy can be lifesaving [23].

### 3.2. Rupture after Dural Opening

Intraoperative rupture of aneurysm is a risk factor associated with the development of intracranial infarct [43]. However, an open skull and dura may better accommodate the pathologically swollen brain during IAR, which in turn may have a beneficial effect on prognosis [44]. After dural opening, IAR mainly occurs during aneurysm dissection, dissection of an adherent artery, or during clip placement [45]. One study revealed that aneurysmal rupture during dissection could be attributed to blunt dissection techniques in 75% of the cases and to sharp subarachnoid dissection in 25% [17]. Other causes of premature rupture are dural and arachnoid opening, hematoma removal, and brain retraction. Management of IAR after dural opening is described below.

#### 3.2.1. Hemodynamic Management

Blood pressure reduction to MAPs of approximately 50 mmHg has been widely advocated in the literature [15, 16, 18, 19, 22]. This may allow for improved surgical exposure as well as a soft aneurysm neck which could be more easily clipped during IAR [15, 16, 22]. However, the MAP of 50–60 mm Hg can be derived from various combinations of systolic and diastolic blood pressure readings and is thus difficult to predict. In addition, this degree of blood pressure reduction is of questionable utility as a means of reducing arterial bleeding. Furthermore, controlled hypotension will have a detrimental effect on cerebral perfusion pressure, which in turn can worsen cerebral ischemia [22]. This is particularly problematic in the setting of ruptured cerebral aneurysm, as these patients may have impaired cerebral autoregulation [46]. Autoregulation may be somewhat preserved in good-grade patients who are operated within 48 hours of ictus; however, the preservation of cerebral autoregulation is not routinely tested in most centers [46, 47]. Thus the maintenance of normotension may be the most appropriate option for these cases [48]. Normovolemia, euglycemia, and electrolyte balance are also important factors which play a crucial role [20, 22]. Transfusion of blood is seldom required; nonetheless, blood should be readily available [49].

Clip application or temporary occlusion in the setting of IAR can at times be exceedingly difficult. However, this procedure may be facilitated by reversible transient complete flow arrest. Adenosine has been used for transient flow arrest, and both its effectiveness and safety have been advocated by many investigators [50, 51]. Adenosine-induced asystole has also been shown to improve circumferential visualization of the aneurysm neck. The recommended starting dose of adenosine is 0.3 to 0.4 mg/kg ideal body weight to achieve approximately 45 seconds of profound systemic hypotension during a remifentanil/low-dose volatile anesthetic, with propofol-induced burst suppression [50]. Larger trials are warranted to provide further information on long term outcomes.

Transient flow arrest via rapid ventricular pacing can also facilitate surgical management of IAR. Rapid ventricular pacing, a technique from interventional cardiology, can be used to induce flow arrest lasting a few seconds. Its role has been the subject of recent investigations in aneurysm surgery, and it has been found to be an effective as well as safe technique for the facilitation of aneurysm surgery [52]. Again, this can provide the surgeon with a soft aneurysm which can be dissected and clipped easily. However, before this method can be considered standard practice, further investigation is required.

#### 3.2.2. Hypothermia

Hypothermia is a potent physiological factor that suppresses the increased cerebral metabolism as well as basal metabolic rate. It acts as a free radical scavenger and stabilizes the neuronal membrane potentials. It thus exerts neuroprotective effects in cerebral ischemia. Many studies have highlighted its potential protective benefits during aneurysm surgeries; however, other studies discourage its routine use [53–57]. Recent reviews have suggested that in good-grade patients, there is no harm in inducing mild hypothermia (32°C–35°C), but nonetheless it is not routinely recommended [56, 57]. For poor-grade patients, evidence has either been insufficient or has shown no benefit. Data is also scanty regarding the role of mild hypothermia initiated during intraoperative rupture, as well as its effect on neurological outcomes. Further investigation is warranted in this respect. The role of hypothermia in coiling procedures has never been investigated and is thus another potential area for research. The effect of hypothermia in open versus closed space procedures (clipping versus coiling) and in preprocedural versus intraprocedural rupture also remains to be determined. In summary, the effect of hypothermia on aneurysmal surgery is confounded by various factors including time of clip application, presence of ruptured aneurysm, grade of SAH, degree of hypothermia, neuroprotective agents, monitoring methods, anesthetic agents, surgeon experience, and finally the intraoperative aneurysm rupture. It will be challenging for future generations of investigators to analyze all contributing factors and present some convincing data on this issue.

#### 3.2.3. Neuroprotection

Prophylactic and therapeutic neuroprotective therapies have been investigated; however, their effectiveness in human subjects is still inconclusive. Both physiologically based (hyperoxygenation, hypothermia, avoidance of hyperthermia and hyperglycemia, hypertension, hemodilution, and hypervolaemia) and pharmacologically based (antifibrinolytic drugs, calcium antagonists, anesthetics, magnesium, erythropoietin, and others) therapies have been explored [58, 59]. Post hoc analysis of Intraoperative Hypothermia for Aneurysm Surgery Trial (IHAST) concluded that supplemental protective drugs used during temporary occlusion have no effect on either short or long term neurological outcomes [60]. Combination of different methods of neuroprotection may be a reasonable option; however, extensive research is still warranted [61]. Further analysis of IHAST data also revealed that nitrous oxide was associated with increased risk of delayed neurological deficits; however, long term neurological outcomes remained unaffected [62]. The above factors primarily pertain to temporary vessel occlusion; however, the effect of each factor during frank intraoperative rupture has never been examined nor is it feasible to conduct a randomized control trial. In the event of IAR, the use of propofol/thiopentone seems to be a reasonable option as these drugs will reduce both CMR and blood pressure [63, 64]. Barbiturates have been found to exert their beneficial effects by mechanisms other than CMR reduction, and administration of high doses to achieve burst suppression may not be required to obtain maximal protection [65]. The role of volatile anesthetic agents including isoflurane and sevoflurane is linked with ischemic preconditioning in animals; however, this has yet to be shown in humans [66]. At the time of intraoperative rupture, volatile agents may be used; however, their use during embolization is not recommended as these agents can increase cerebral blood volume (ICP) and worsen cerebral ischemia.

#### 3.2.4. Surgical Management

Temporary occlusion is one of the greatest advancements in aneurysm surgery [67–69]. Not only does it assist in proper aneurysm clipping, but it decreases the incidence of IAR. Multiple variables have an effect on the success of temporary occlusion, including age, grade of SAH, duration, location of aneurysm, and frequency [70, 71]. In addition, type of cerebral protection, hemodynamic set points, temperature, and type of neurophysiological monitoring may have an effect on outcomes in relation to temporary occlusion, though the literature is presently inconclusive [72–74].

There are fundamental differences in the pathophysiological mechanisms, neuroradiological findings, and postoperative outcomes in elective neurosurgical patients experiencing brain herniation as compared to other surgical populations. Intraoperative brain herniation secondary to extra-axial subarachnoid or intraventricular hemorrhage has a substantially better outcome compared to herniation caused by intraparenchymal hemorrhage. Seldom expeditious abandonment of the procedure and closure of the cranium may also contribute to the often very satisfactory clinical outcome.

#### 3.2.5. Neurointerventional Management

Preprocedural perforation during neurointerventional procedures (like angiography, induction of anesthesia) usually requires urgent surgical management including emergency ventriculostomy and/or decompressive hemicraniectomy [23]. Intraprocedural rupture can be managed with neurointerventional methods including partial or complete packing of aneurysmal sac with coils.

## 4. Discussion

**Figure 1:**
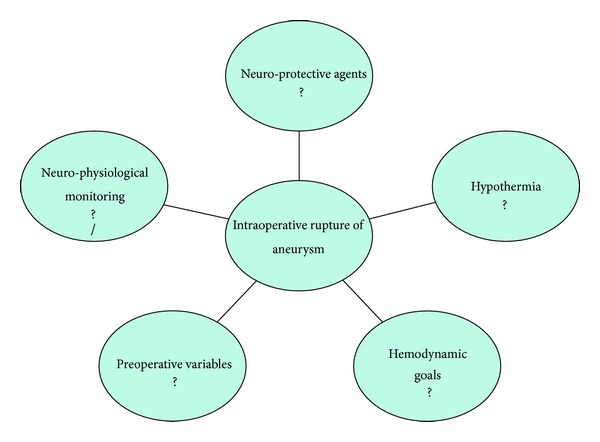
Controversial issues related to the management of IAR.

The purpose of this review is to present current knowledge, evidence, and practices related to the management of IAR. However, there exist some important controversies (Table 3, Figure 1) pertaining to the management of IAR [20, 22, 41, 75].

The *first controversy* relates to management of preexisting factors that affect risk of IAR, including hypertension, induction of anesthesia, factors related to TMPG, and presence of various comorbidities [10–22]. Hypertension is known to have an effect on the occurrence of IAR, and associated features such as disease duration and treatment compliance may also be of some importance. Furthermore, preoperative omission of antihypertensive medications coupled with increased sympathetic stimulation at the time of surgery may also play a role in the occurrence of IAR. However, these concerns are of primarily theoretical concern at present as the data has been inconclusive thus far. Induction of anesthesia has also been highlighted as a potential cause for IAR in a few studies, related to both sympathetic stimulation (hypertensive response) and airway stimulation (cough/gag) [15, 16]. However, IAR during induction may also be influenced by the presence of other cofactors such as timing of surgery, aneurysmal sac thickness, aneurysm size and location, and patient comorbidities [11–13, 15, 16]. Appropriate optimization strategies related to these variables have yet to be determined; further research in both the basic and clinical sciences is required. Irrespective of all the factors discussed, anesthesiologists should take all possible measures to prevent excessive sympathetic stimulation throughout surgery, particularly during periods of increased vulnerability (intubation, surgical stimuli, extubation, etc.).

The reduction of TPMG prior to craniotomy and dural opening, via osmotherapy and hyperventilation, has been proposed as a means of reducing risk of IAR. However, the relationship between TPMG control and reduction of IAR risk is not clear and remains a largely theoretical consideration [20, 22].

Certain conditions such as COPD, coronary artery disease, hyperlipidemia, and smoking are linked with IAR; however, the feasibility of their optimization before surgery is still a question [9]. Similarly, smoking is not only related to many adverse effects intraoperatively (increased airway sensitivity, carboxyhemoglobin level) but imposes great risks postoperatively (laryngospasm, coughing, gagging, and infection). Smoking cessation appears to reduce the risk of aneurysmal rupture; however, there is no consensus on the timing of smoking cessation in patients undergoing surgery [9]. There is also conflicting evidence regarding the continuation of surgery in cases of IAR at induction of anesthesia; however, rescue clipping has shown favorable outcomes in this situation [15, 16].

The *second controversy* pertains to diagnostic techniques, in particular neurophysiological monitoring. The role of EEG and evoked potentials in detection of ischemia related to IAR depends upon many factors including presence or absence of burst suppression, use of volatile agents/muscle relaxants, hemodynamic parameters, and temperature [24, 25]. The ischemia produced outside the defined pathways of these evoked potentials are another area of concern [26–32]. EEG is mainly a global indicator of cerebral ischemia; its role in the detection of posterior fossa ischemia is limited. The role of NPMs in endovascular management remains limited due to concern about the possibility of interference from EEG electrodes [76]. There also exists a variable time lag of up to a few minutes between changes detected by the monitors and the development of ischemia [24, 25]. However, there is no other available monitoring technique that could assess the effectiveness of NPM for detection of cerebral ischemia.

*Thirdly*, hemodynamic goals, hypothermia, pharmacologic neuroprotection, and burst suppression are all factors which require further study to elucidate their effects on patient outcomes. Hemodynamic goals during different procedures (clipping versus coiling), as well as during various phases of surgery (preprocedural versus intraprocedural), are still not well defined and remain a matter of conflict [15, 16, 20, 22, 23]. Induced hypotension has been shown to decrease brain swelling during IAR prior to dural opening, while other measures such as osmotherapy and steroids have not. This suggests that brain edema is not the primary pathophysiological mechanism in these situations [15, 16]. The aggravation of ongoing cerebral ischemia during IAR is a potential side effect of induced hypotension [15, 16, 22]. The normotension or 20% decrease from baseline may be the optimal choice in these situations [48]. On the other hand, intraprocedural rupture with signs of ischemia sometimes warrants induced hypertension. The role of neuroprotective strategies has been mentioned during the time of temporarily clipping; however, its usefulness in case for IAR remains a matter of ongoing investigation [67–74]. Similarly, the effect of hypothermia in these cases has yet to be determined [56, 57]. Furthermore, the effect of hypothermia in IAR cases with different grades (good versus poor grade) may be differential and future attention is required [56, 57]. Different management strategies in relation to IAR during clipping versus coiling also need further attention. A thorough knowledge and understanding of these current areas of controversy would open the gate for future guidelines and standards of care.

## 5. Future Directions

The goal of future therapy will remain focused on the development of preventive techniques and will also involve strategies to improve outcomes in IAR when it still occurs. A thorough understanding of various factors, including anesthetic, surgical, neurointerventional, and neurophysiological factors, will play a pivotal role in the development of future therapies. Preventive strategies should focus on smoking cessation as well as optimization of various comorbidities and cofactors [9]. Newer protective agents in neurointerventional procedures are being investigated and are at least partly based on ischemic preconditioning. Pharmacological aneurysm stabilization is an area of recent interest, and research in animal models is ongoing. Tetracycline derivatives have shown some promising results [77]. Much more research should be oriented towards hypothermia and other neuroprotective strategies. Hemodynamic goals should be better defined in relation to IAR prevention, in balance with maintenance of adequate cerebral perfusion pressure.

## 6. Conclusion

As part of the overarching goals of enhancing patient care and improving neurological outcomes, it is necessary to consider both the usefulness and fallacies of current practices in IAR management. Further research in basic sciences is still required in order to improve understanding of the underlying pathophysiological mechanisms. Future studies will decide whether or not current management techniques should be adopted as the standard of care.

## Figures and Tables

**Table 1:** Intraoperative factors contributing to intraoperative aneurysm rupture.

Factors	Controversies
Hypertension	Upper limit of blood pressure
	Poorly controlled BP/controlled BP
	Chronic/acute hypertension
Anesthetic factors	Sympathetic responses (intubation/extubation)
	Coughing/gagging
ICP	Sudden decrease in ICP during
	hyperventilation, use of large dose mannitol, and CSF drain
Maneuvers	Valsalva, application of PEEP (upper limit)
Comorbidities	COPD, CAD, and hyperlipidemia

**Table 2:** Diagnosis of IAR.

Method	Findings
(1) Clinical	Hypertension, bradycardia, and arrhythmias
	Blown pupil
(2) Surgical	Increase ooze from surgical incision
	Brain bulge, Hematoma
(3) Monitoring	
ICP	Sudden rise in ICP, presence of pathological waves
TCD	No diastolic flow to reversal of diastolic flow
Cerebral oximetry	Sudden decrease in values
Neurophysiological monitoring	
EEG	Suppression of electrical activity
	Burst suppression, complete silence
SSEP	50% reduction in amplitude and/or 10% increase in latency
MEP	Increase in stimulus threshold
	Decrease in amplitude
BEAP	Increase in latency (more than 1 msec) in wave V
(4) Radiological	Contrast-dye extravasation
	Prolongation of dye transit time
	Slowing/flow arrest ICA, flow reversal to ECA

**Table 3:** Controversies in the management of IAR.

Management strategy	Controversies
IAR at anesthesia induction	Role of rescue clipping versus cancellation of surgery
Preoperative variables	Effect on IAR, role of optimization, and smoking cessation (minimal time)
Neurophysiological monitoring	Role of EEG during burst suppression during IAR
	Role of SSEP to detect ischemia outside the somatosensory pathway
Clip placement (temporarily)	Effect on outcome of induced hypertension with or without burst suppression
	Effect of normotension
Hemodynamic	Hypotension or normotension
	Goal of MAP during IAR
	Role of adenosine and ventricular pacing
Hypothermia	Mild to moderate hypothermia (time and duration)
	In good-grade patients/poor-grade patients
	In coiling patients
	With or without neuroprotective agents
Neuroprotection	Role of different agents on outcome
	With or without hypothermia
	Thiopental and requirement of burst suppression
Hyperventilation	Values at the time of IAR
	Time and duration
	Role of measuring cerebral oxygenation

## References

[1] Vlak MH, Algra A, Brandenburg R, Rinkel GJ (2011). Prevalence of unruptured intracranial aneurysms, with emphasis on sex, age, comorbidity, country, and time period: a systematic review and meta-analysis. *The Lancet Neurology*.

[2] Brown RD (2010). Unruptured intracranial aneurysms. *Seminars in Neurology*.

[3] Bederson JB, Connolly ES, Batjer HH (2009). Guidelines for the management of aneurysmal subarachnoid hemorrhage: a statement for healthcare professionals from a special writing group of the stroke council, American Heart Association. *Stroke*.

[4] Zacharia BE, Ducruet AF, Hickman ZL (2011). Technological advances in the management of unruptured intracranial aneurysms fail to improve outcome in New York State. *Stroke*.

[5] Caner B, Hou J, Altay O, Fuj M, Zhang JH (2012). Transition of research focus from vasospasm to early brain injury after subarachnoid hemorrhage. *Journal of Neurochemistry*.

[6] Rowland MJ, Hadjipavlou G, Kelly M, Westbrook J, Pattinson KT (2012). Delayed cerebral ischaemia after subarachnoid haemorrhage: looking beyond vasospasm. *British Journal of Anaesthesia*.

[7] Leipzig TJ, Morgan J, Horner TG, Payner T, Redelman K, Johnson CS (2005). Analysis of intraoperative rupture in the surgical treatment of 1694 saccular aneurysms. *Neurosurgery*.

[8] Nanda A, Willis BK, Vannemreddy PS (2002). Selective intraoperative angiography in intracranial aneurysm surgery: intraoperative factors associated with aneurysmal remnants and vessel occlusions. *Surgical Neurology*.

[9] Elijovich L, Higashida RT, Lawton MT (2008). Predictors and outcomes of intraprocedural rupture in patients treated for ruptured intracranial aneurysms: the CARAT study. *Stroke*.

[10] Inagawa T (2010). Risk factors for the formation and rupture of intracranial saccular aneurysms in Shimane, Japan. *World Neurosurgery*.

[11] Clarke M (2008). Systematic review of reviews of risk factors for intracranial aneurysms. *Neuroradiology*.

[12] Dubow J, Fink ME (2011). Impact of hypertension on stroke. *Current Atherosclerosis Reports*.

[13] Nahed BV, DiLuna ML, Morgan T (2005). Hypertension, age, and location predict rupture of small intracranial aneurysms. *Neurosurgery*.

[14] Taylor CL, Yuan Z, Selman WR, Ratcheson RA, Rimm AA (1995). Cerebral arterial aneurysm formation and rupture in 20,767 elderly patients: hypertension and other risk factors. *Journal of Neurosurgery*.

[15] Tsementzis SA, Hitchcock ER (1985). Outcome from “rescue clipping” of ruptured intracranial aneurysms during induction anaesthesia and endotracheal intubation. *Journal of Neurology Neurosurgery & Psychiatry*.

[16] Beatty RA (1990). Intraoperative aneurysms rupture during the predissection stage. *Journal of Neurology Neurosurgery & Psychiatry*.

[17] Batjer H, Samson D (1986). Intraoperative aneurysmal rupture: Incidence, outcome, and suggestions for surgical management. *Neurosurgery*.

[18] Dangor AA, Lam AM (1998). Anesthesia for cerebral aneurysm surgery. *Neurosurgery Clinics of North America*.

[19] Herrick IA, Gelb AW (1992). Anesthesia for intracranial aneurysm surgery. *Journal of Clinical Anesthesia*.

[20] Connolly JS, Rabinstein AA, Carhuapoma JR (2012). Guidelines for the management of aneurysmal subarachnoid hemorrhage: a guideline for healthcare professionals from the American Heart Association/American Stroke Association. *Stroke*.

[21] Heuer GG, Smith MJ, Elliott JP, Winn HR, Leroux PD (2004). Relationship between intracranial pressure and other clinical variables in patients with aneurysmal subarachnoid hemorrhage. *Journal of Neurosurgery*.

[22] Priebe H-J (2007). Aneurysmal subarachnoid haemorrhage and the anaesthetist. *British Journal of Anaesthesia*.

[23] Sharma DP, Singh D, Jagetia A, Singh H, Tandon M, Ganjoo P (2011). Intra procedure rupture of intracranial aneurysm during endovascular coiling: neurosurgeons' experience and review of the literature. *Neurology India*.

[24] Bacigaluppi S, Fontanella M, Manninen P, Ducati A, Tredici G, Gentili F (2012). Monitoring techniques for prevention of procedure-related ischemic damage in aneurysm surgery. *World Neurosurg*.

[25] Sloan MA (2006). Prevention of ischemic neurologic injury with intraoperative monitoring of selected cardiovascular and cerebrovascular procedures: roles of electroencephalography, somatosensory evoked potentials, transcranial Doppler, and near-infrared spectroscopy. *Neurologic Clinics*.

[26] Schick U, Döhnert J, Meyer J-J, Vitzthum H-E (2005). Effects of temporary clips on somatosensory evoked potentials in aneurysm surgery. *Neurocritical Care*.

[27] Buchthal A, Belopavlovic M, Mooij JJA (1988). Evoked potential monitoring and temporary clipping in cerebral aneurysm surgery. *Acta Neurochirurgica*.

[28] Buchthal A, Belopavlovic M (1988). Somatosensory evoked potentials in cerebral aneurysm surgery. *Klinische Wochenschrift*.

[29] Kidooka M, Nakasu Y, Watanabe K, Matsuda M, Handa J (1987). Monitoring of somatosensory-evoked potentials during aneurysm surgery. *Surgical Neurology*.

[30] Lopéz JR, Chang SD, Steinberg GK (1999). The use of electrophysiological monitoring in the intraoperative management of intracranial aneurysms. *Journal of Neurology, Neurosurgery, & Psychiatry*.

[31] Florence G, Guerit J-M, Gueguen B (2004). Electroencephalography (EEG) and somatosensory evoked potentials (SEP) to prevent cerebral ischaemia in the operating room. *Neurophysiologie Clinique*.

[32] Krayenbühl N, Sarnthein J, Oinas M, Erdem E, Krisht AF (2011). MRI-validation of SEP monitoring for ischemic events during microsurgical clipping of intracranial aneurysms. *Clinical Neurophysiology*.

[33] Eng CC, Lam AM, Byrd S, Newell DW (1993). The diagnosis and management of a perianesthetic cerebral aneurysmal rupture aided with transcranial Doppler ultrasonography. *Anesthesiology*.

[34] Park J, Woo H, Kim GC (2012). Diagnostic usefulness of intraoperative ultrasonography for unexpected severe brain swelling in ultra-early surgery for ruptured intracranial aneurysms. *Acta Neurochirurgica*.

[35] Rozet I, Newell DW, Lam AM (2006). Intraoperative jugular bulb desaturation during acute aneurysmal rupture. *Canadian Journal of Anesthesia*.

[36] Schnell O, Morhard D, Holtmannspötter M, Reiser M, Tonn J-C, Schichor C (2012). Near-infrared indocyanine green videoangiography (ICGVA) and intraoperative computed tomography (iCT): are they complementary or competitive imaging techniques in aneurysm surgery?. *Acta Neurochirurgica*.

[37] Schichor C, Rachinger W, Morhard D (2010). Intraoperative computed tomography angiography with computed tomography perfusion imaging in vascular neurosurgery: feasibility of a new concept. *Journal of Neurosurgery*.

[38] Pickard JD, Lovick AH, Read DH (1986). Evidence of brain engorgement as the initial cause of brain swelling following intraoperative aneurysm rupture in man after subarachnoid haemorrhage. *Acta Physiologica Scandinavica*.

[39] Horowitz MB, Crammond D, Balzer J, Jungreis C, Kassam AB (2003). Aneurysm rupture during endovascular coiling: effects on cerebral transit time and neurophysiologic monitoring and the benefits of early ventriculostomy: case report. *Minimally Invasive Neurosurgery*.

[40] Mahaney KB, Todd MM, Bayman EO, Torner JC (2012). Acute postoperative neurological deterioration associated with surgery for ruptured intracranial aneurysm: incidence, predictors, and outcomes. *Journal of Neurosurgery*.

[41] Ausman JI, Diaz FG, Malik GM, Fielding AS, Son CS (1985). Current management of cerebral aneurysms: is it based on facts or myths?. *Surgical Neurology*.

[42] Yuguang L, Tao J, Meng L (2003). Rerupture of intracranial aneurysms during cerebral angiography. *Journal of Clinical Neuroscience*.

[43] Umredkar A, Gupta SK, Khandelwal N (2010). Intracerebral infarcts following clipping of intracranial aneurysms: Incidence, clinical correlation and outcome. *British Journal of Neurosurgery*.

[44] Sandalcioglu IE, Schoch B, Regel JP (2004). Does intraoperative aneurysm rupture influence outcome? Analysis of 169 patients. *Clinical Neurology and Neurosurgery*.

[45] Lawton MT, Du R (2005). Effect of the neurosurgeon's surgical experience on outcomes from intraoperative aneurysmal rupture. *Neurosurgery*.

[46] Cossu M, Gennaro S, Rossi A, Balestrero MA, Cella F, Viale GL (1999). Autoregulation of cortical blood flow during surgery for ruptured intracranial aneurysms. *Journal of Neurosurgical Sciences*.

[47] Dernbach PD, Little JR, Jones SC, Ebrahim ZY (1988). Altered cerebral autoregulation and CO_2_ reactivity after aneurysmal subarachnoid hemorrhage. *Neurosurgery*.

[48] Giannotta SL, Oppenheimer JH, Levy ML, Zelman V (1991). Management of intraoperative rupture of aneurysm without hypotension. *Neurosurgery*.

[49] Le Roux PD, Elliott JP, Winn HR (2001). Blood transfusion during aneurysm surgery. *Neurosurgery*.

[50] Bendok BR, Gupta DK, Rahme RJ (2011). Adenosine for temporary flow arrest during intracranial aneurysm surgery: a single-center retrospective review. *Neurosurgery*.

[51] Luostarinen T, Takala RSK, Niemi TT (2010). Adenosine-induced cardiac arrest during intraoperative cerebral aneurysm rupture. *World Neurosurgery*.

[52] Saldien V, Menovsky T, Rommens M (2012). Rapid ventricular pacing for flow arrest during cerebrovascular surgery: revival of an old concept. *Neurosurgery*.

[53] Mackensen GB, McDonagh DL, Warner DS (2009). Perioperative hypothermia: use and therapeutic implications. *Journal of Neurotrauma*.

[54] Seule MA, Muroi C, Mink S, Yonekawa Y, Keller E (2009). Therapeutic hypothermia in patients with aneurysmal subarachnoid hemorrhage, refractory intracranial hypertension, or cerebral vasospasm. *Neurosurgery*.

[55] Cottrell JE, Hartung J (2004). Cool it on cooling—at least during aneurysm surgery. *Journal of Neurosurgical Anesthesiology*.

[56] Zhao ZX, Wu C, He M (2012). A systematic review of clinical outcomes, perioperative data and selective adverse events related to mild hypothermia in intracranial aneurysm surgery. *Clinical Neurology and Neurosurgery*.

[57] Li LR, You C, Chaudhary B (2012). Intraoperative mild hypothermia for postoperative neurological deficits in intracranial aneurysm patients. *Cochrane Database of Systematic Reviews*.

[58] Frietsch T, Kirsch JR (2004). Strategies of neuroprotection for intracranial aneurysms. *Best Practice & Research Clinical Anaesthesiology*.

[59] Hill MD, Martin RH, Mikulis D (2012). Safety and efficacy of NA-1 in patients with iatrogenic stroke after endovascular aneurysm repair (ENACT): a phase 2, randomised, double-blind, placebo-controlled trial. *The Lancet Neurology*.

[60] Hindman BJ, Bayman EO, Pfisterer WK, Torner JC, Todd MM, IHAST Investigators (2010). No association between intraoperative hypothermia or supplemental protective drug and neurologic outcomes in patients undergoing temporary clipping during cerebral aneurysm surgery: findings from the intraoperative hypothermia for aneurysm surgery trial. *Anesthesiology*.

[61] Schaller B, Graf R, Jacobs AH (2003). Ischaemic tolerance: a window to endogenous neuroprotection?. *The Lancet*.

[62] Pasternak JJ, Mcgregor DG, Lanier WL (2009). Effect of nitrous oxide use on long-term neurologic and neuropsychological outcome in patients who received temporary proximal artery occlusion during cerebral aneurysm clipping surgery. *Anesthesiology*.

[63] McDermott MW, Durity FA, Borozny M, Mountain MA, Piepgras DG (1989). Temporary vessel occlusion and barbiturate protection in cerebral aneurysm surgery. *Neurosurgery*.

[64] Erickson KM, Pasternak JJ, Weglinski MR, Lanier WL (2009). Temperature management in studies of barbiturate protection from focal cerebral ischemia: systematic review and speculative synthesis. *Journal of Neurosurgical Anesthesiology*.

[65] Schmid-Elsaesser R, Schröder M, Zausinger S, Hungerhuber E, Baethmann A, Reulen H-J (1999). EEG burst suppression is not necessary for maximum barbiturate protection in transient focal cerebral ischemia in the rat. *Journal of the Neurological Sciences*.

[66] Schifilliti D, Grasso G, Conti A, Fodale V (2010). Anaesthetic-related neuroprotection. *CNS Drugs*.

[67] Bellotti C, Oliveri G, Allegra G (1998). Temporary clipping in the surgery of endocranial aneurysms. *Journal of Neurosurgical Sciences*.

[68] Taylor CL, Selman WR (1998). Temporary vascular occlusion during cerebral aneurysm surgery. *Neurosurgery Clinics of North America*.

[69] Taylor CL, Selman WR, Kiefer SP, Ratcheson RA (1996). Temporary vessel occlusion during intracranial aneurysm repair. *Neurosurgery*.

[70] Ogilvy CS, Carter BS, Kaplan S, Rich C, Crowell RM (1996). Temporary vessel occlusion for aneurysm surgery: risk factors for stroke in patients protected by induced hypothermia and hypertension and intravenous mannitol administration. *Journal of Neurosurgery*.

[71] Samson D, Batjer HH, Bowman G (1994). A clinical study of the parameters and effects of temporary arterial occlusion in the management of intracranial aneurysms. *Neurosurgery*.

[72] Zhou LF, Li SQ, Yang ML (1993). Temporary arterial occlusion during intracranial aneurysm surgery. *Chinese Medical Journal*.

[73] Ogawa A, Sato H, Sakurai Y, Yoshimoto T (1991). Limitation of temporary vascular occlusion during aneurysm surgery: study by intraoperative monitoring of cortical blood flow. *Surgical Neurology*.

[74] Jabre A, Symon L (1987). Temporary vascular occlusion during aneurysm surgery. *Surgical Neurology*.

[75] Ausman JI, Diaz FG, Malik GM, Andrews BT, McCormick PW, Balakrishnan G (1989). Management of cerebral aneurysms: further facts and additional myths. *Surgical Neurology*.

[76] Chen L, Spetzler RF, McDougall CG, Albuquerque FC, Xu B (2011). Detection of ischemia in endovascular therapy of cerebral aneurysms: a perspective in the era of neurophysiological monitoring. *Neurosurgical Review*.

[77] Nussbaum ES, Janjua TM, Defillo A, Sinner P, Zelensky A (2009). Perioperative use of recombinant factor VII to prevent intraoperative aneurysm rupture in high risk patients: a preliminary safety evaluation. *Neurocritical Care*.

